# *Aggregatibacter actinomycetemcomitans* and *Filifactor alocis*: Two exotoxin-producing oral pathogens

**DOI:** 10.3389/froh.2022.981343

**Published:** 2022-08-15

**Authors:** Hazel Ozuna, Ian Snider, Georgios N. Belibasakis, Jan Oscarsson, Anders Johansson, Silvia M. Uriarte

**Affiliations:** ^1^Department of Microbiology and Immunology, School of Medicine, University of Louisville, Louisville, KY, United States; ^2^Department of Biology, School of Arts and Sciences, University of Louisville, Louisville, KY, United States; ^3^Department of Dental Medicine, Karolinska Institutet, Huddinge, Sweden; ^4^Department of Odontology, Umeå University, Umeå, Sweden; ^5^Department of Oral Immunology and Infectious Diseases, School of Dentistry, University of Louisville, Louisville, KY, United States

**Keywords:** periodontitis, *Filifactor alocis*, *Aggregatibacter actinomycetemcomitans*, virulence factors, chronic inflammation

## Abstract

Periodontitis is a dysbiotic disease caused by the interplay between the microbial ecosystem present in the disease with the dysregulated host immune response. The disease-associated microbial community is formed by the presence of established oral pathogens like *Aggregatibacter actinomycetemcomitans* as well as by newly dominant species like *Filifactor alocis*. These two oral pathogens prevail and grow within the periodontal pocket which highlights their ability to evade the host immune response. This review focuses on the virulence factors and potential pathogenicity of both oral pathogens in periodontitis, accentuating the recent description of *F. alocis* virulence factors, including the presence of an exotoxin, and comparing them with the defined factors associated with *A. actinomycetemcomitans*. In the disease setting, possible synergistic and/or mutualistic interactions among both oral pathogens might contribute to disease progression.

## Introduction

Periodontitis is a multifactorial irreversible chronic inflammatory disease that affects the supporting structure of the teeth. Recent reports by the Centers for Disease Control and Prevention (CDC) show that 42.2% of adults 30 years and older develop some form of periodontitis [1]. Poor oral hygiene is the most common cause associated with periodontitis, but other factors such as age, gender, socioeconomic, and education status increase the risk to develop the disease [2]. Other risk factors such as smoking, diabetes, medications that cause dry mouth, stress, and genetics [1] can affect the oral cavity homeostasis and have a direct or indirect impact on the oral microbiome composition. This in turn promotes changes in the abundance and homeostatic relationships within the polymicrobial communities resulting in a dysbiotic ecosystem in disease. The disruption of tissue homeostasis is accompanied by microbial shifts or dysbiosis from indigenous symbionts (commensal bacteria) to predominantly pathogenic bacteria. The new state of polymicrobial dysbiosis promotes a dysregulated inflammatory state in the host that drives disease progression. Long studied microorganisms such as *Porphyromonas gingivalis, Tannerella forsythia, Treponema denticola*, and *Aggregatibacter actinomycetemcomitans* are now well-established “periodontal pathogens”, with evidenced involvement in disease initiation and progression. In the last decade, the advance in high throughput technology allowed us to obtain an in-depth characterization of the complexity of the oral microbiome both in health and in disease [3, 4]. As a result, several microorganisms were identified with high prevalence in disease sites compared to healthy sites, including *Filifactor alocis*, a newly appreciated microbial species.

**Figure 1:**
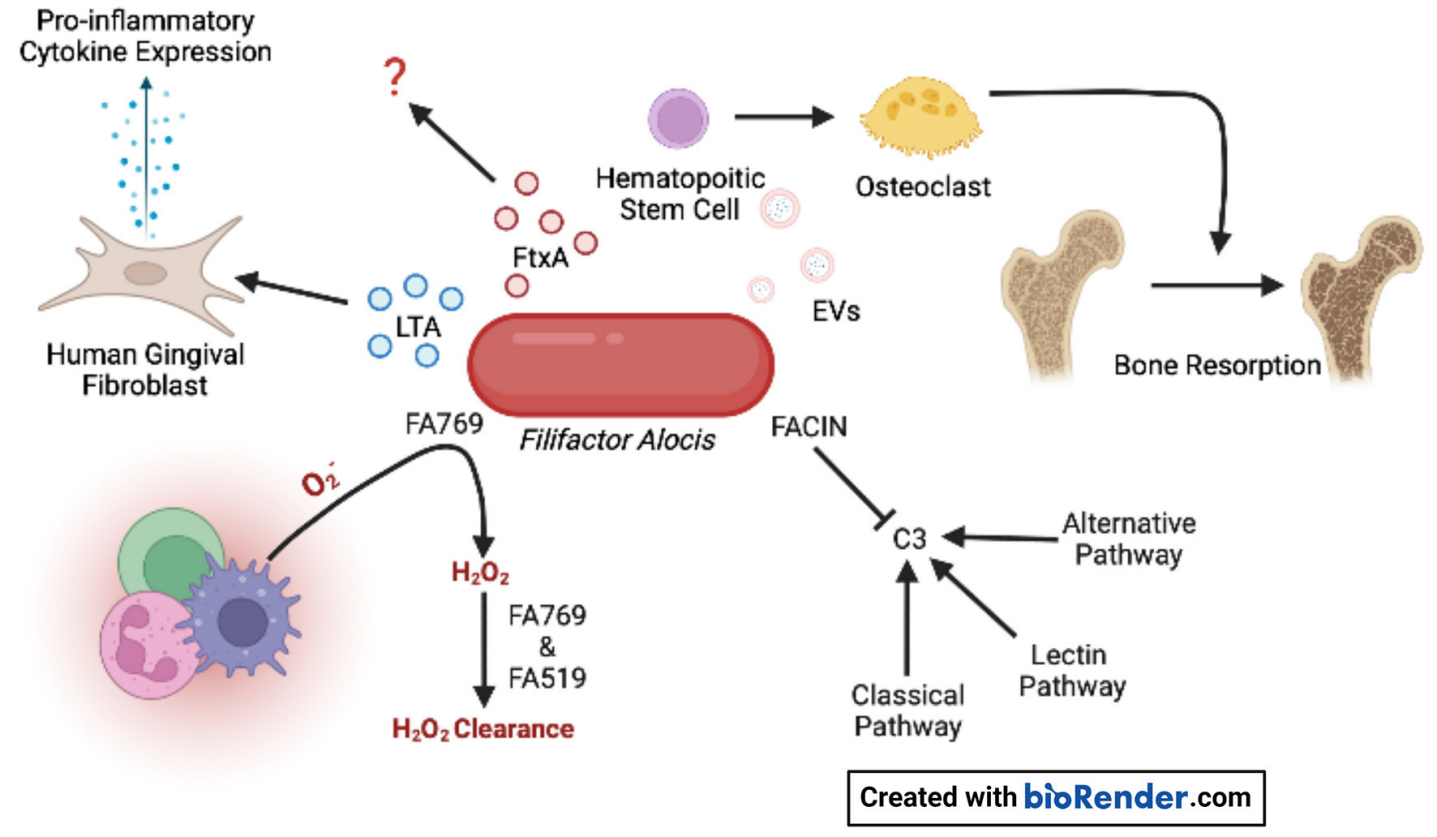
*F. alocis* virulence factors. Oxidative resistance is achieved by the conversion of superoxide generated by innate immune cells to H_2_O_2_ by FA769. The proteins FA769 and FA519 are associated with H_2_O_2_-induced stress resistance. *F. alocis* complement inhibitor (FACIN) binds complement component 3 (C3) which is essential to all three complement pathways. Extracellular vesicles (EVs) contain lipoproteins that stimulate osteoclastogenesis in committed osteoclast precursors *via* TLR2 which contributes to bone resorption. The novel RTX exotoxin, FtxA, is found in 60% of clinical isolates, but its biological effects are unknown. Lipoteichoic acid (LTA) induces the expression of pro-inflammatory cytokines by human gingival fibroblasts.

*A. actinomycetemcomitans* is a non-motile gram-negative facultative anaerobe of the *Pasteurellaceae* family [5–8], and is known to contribute to gingival tissue inflammation, destruction, and bone resorption by expressing several virulence factors (Supplementary Table S1) such as cytolethal distending toxin (Cdt), leukotoxin A (LtxA) of the Repeats-In-Toxins (RTX) family of bacterial toxins, and collagenase [9–15]. On the other hand, *F. alocis* is a gram-positive anaerobic rod, and characterization of the organism's pathogenic credentials is still in its infancy. Some initial descriptions of the potential virulence factors of *F. alocis* include the presence of a moonlight surface protein that binds to and inhibits the complement component 3 (C3), a key step of the complement activation cascade; two enzymes that might provide oxidative stress resistance; and an exotoxin of the RTX family with an unknown biological function (Figure 1). Some recent reports describe that the presence of *F. alocis* increases *A. actinomycetemcomitans* total biomass when in co-infections with *Veillonella* sp [16, 17]. In this mini-review, we describe the virulence factors associated with *A. actinomycetemcomitans* and *F. alocis*, which are uniquely found in those species among all members of the oral microbial community.

## Periodontitis: Lessons learned from established and emerging periodontal pathogens

**Figure 2:**
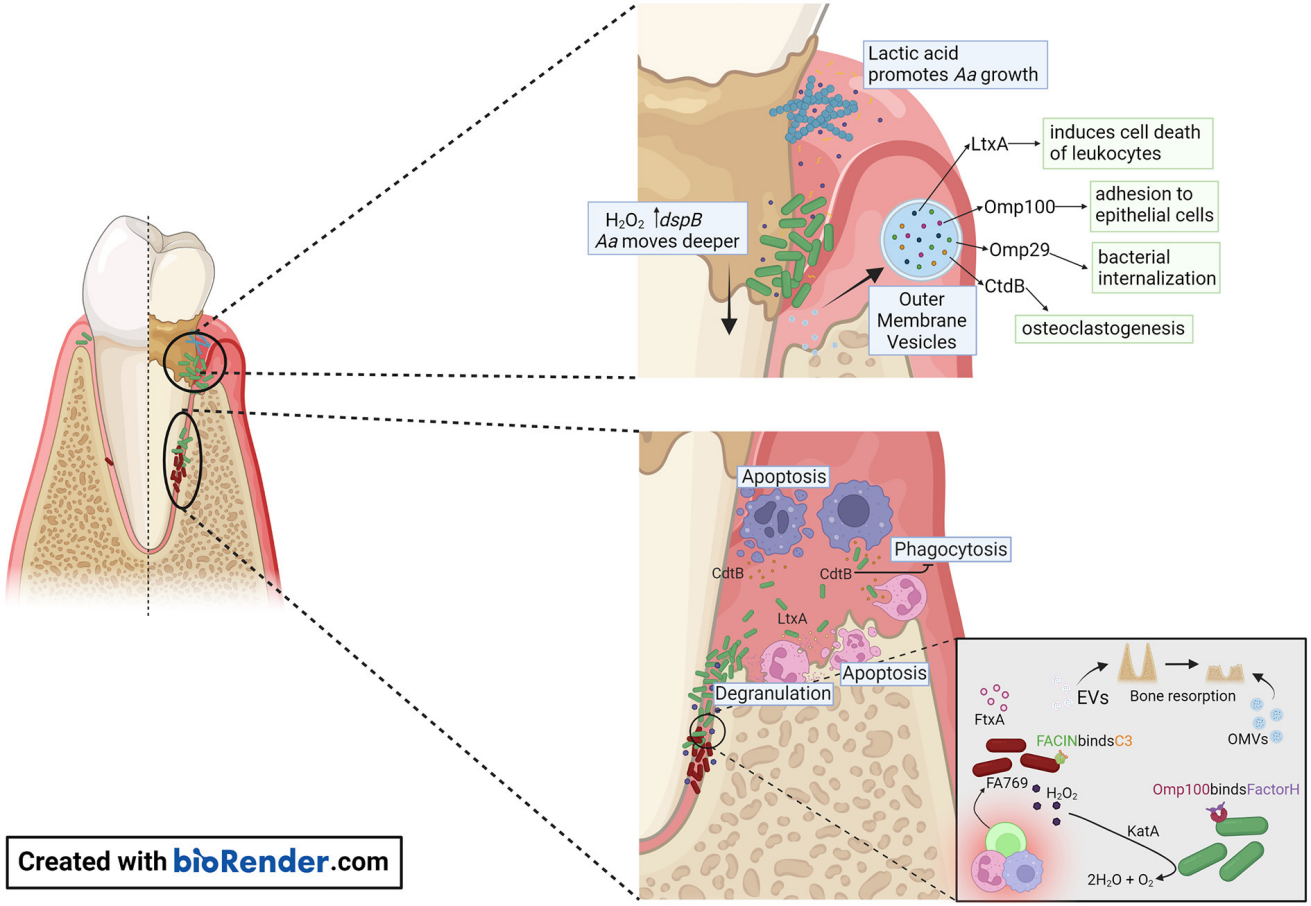
Proposed model of *A. actinomycetemcomitans* and *F. alocis* interactions with the host within the gingival pocket. In the early stages of periodontitis, *A. actinomycetemcomitans* makes use of lactic acid produced by *Streptococcus sp*. as a nutrient to increase its numbers. *A. actinomycetemcomitans* releases OMVs that are packed with outer membrane proteins (Omp) OmpA1, Omp 100, and the virulence factors CdtB, and LtxA. Omp100 mediates initial adhesion of the bacterium, and OmpA1 binds to its putative ligand on gingival epithelial cells and induces F-actin rearrangements resulting in *A. actinomycetemcomitans* cells being internalized. Production of H_2_O_2_ by *Streptococcus sp*. causes *A. actinomycetemcomitans* to migrate deeper in the gingival pocket, where the bacterial cells are exposed to the host immune response. The release of CtdB in this environment inhibits phagocytosis and LtxA release by *A. actinomycetemcomitans* will promote neutrophil degranulation or cell death when present at high concentrations. The release of OMVs by *A. actinomycetemcomitans* or EVs by *F. alocis* might contribute to the pathogenicity of these organisms by evasion of the host immune response and promoting bone resorption.

Subgingival plaque samples collected from periodontitis patients and periodontitis-free individuals differ from each other [18]. *P. gingivalis, T. forsythia*, and *T. denticola*, the so-called red complex, have shown the strongest association with periodontal disease [19]. Deepened periodontal pockets with an anaerobic environment, inflammatory conditions, and large access to substrates originating from tissue destruction all favor the growth of these pathogens and pathobionts. These gram-negative anaerobe pathobionts express virulence factors with the capacity to cause an imbalance in the host inflammatory response [20]. If these bacteria contribute to the degenerative process in periodontitis or if they are a result of the unique ecological niche in a periodontal pocket, it has not been fully investigated [21]. The study of the microbial composition shift from periodontally healthy toward disease onset can contribute to answer this challenging question. Longitudinal studies examined periodontally healthy adolescents that at baseline show that the presence of *A. actinomycetemcomitans* in the subgingival plaque is significantly associated with disease onset [22–25]. The prevalence of this bacterium varies on age, geographic origin, and periodontal status of the examined population [26]. A high intra-species genetic diversity exists, which resulted in the generation of highly virulent as well as harmless variants of this bacterium [27]. The most well-known virulent variant of *A. actinomycetemcomitans* is the JP2 genotype. This genotype expresses a high amount of LtxA and is often detected in young individuals with periodontitis [28, 29]. Interestingly, it has been shown that young individuals that carry *A. actinomycetemcomitans* in their subgingival plaque have an increased risk to develop attachment loss if *F. alocis* is detected in the same sample [17]. Based on these reports we propose a model in which *A. actinomycetemcomitans* initiates the degenerative process in the periodontium that creates an anaerobic environment attractive for translocation of *F. alocis* (Figure 2). In addition, it could be speculated that *F. alocis* manipulation of innate immune cells, like neutrophils, interferes with *A. actinomycetemcomitans* LtxA-induced inflammatory cell death [13, 30].

More recently the anaerobic gram-positive bacterium *F. alocis* has attracted interest in the etiology and pathogenesis of periodontitis [31]. This bacterium is often detected in periodontal pockets of different individuals [32, 33]. The role of *F. alocis* in the pathogenicity of periodontitis is still not known, however, recent studies indicate a capacity to dysregulate the innate immune response [34]. To begin to characterize *F. alocis'* potential virulence factors, we screened the whole genome of the *F. alocis* reference strain ATCC 35896 (also known as CCUG 47790), for novel identification and deeper characterization of virulence elements. We discovered that the reference strain encodes a hitherto unrecognized RTX toxin member, which we designated as “FtxA” for consistency with the nomenclature of other RTX toxin-gene encoding operons [35]. We have used ATCC 35896, and our clinical collection of nine additional *F. alocis* strains, isolated from different oral infections, to further characterize the FtxA protein, and whether the gene encoding it may be conserved in the phylogenetic lineage(s) of *F. alocis*, and hence might represent a candidate diagnostic marker for more virulent strains. According to PCR, *ftxA* was encoded by five of the ten tested strains [35]. To corroborate the PCR results, and for subsequent multi-locus sequence typing (MLST), all nine strains that had been isolated at the clinical laboratory were then subject to whole-genome sequencing. Extraction of the genome sequence data essentially confirmed the PCR findings, with highly conserved FtxA protein sequences, encoded in apparent *ftxABD* operons. However, one of the *ftxA*-negative strains according to PCR, 854G-16U, was found to encode an FtxA homolog. Relative to the ATCC 35896 FtxA protein, it was only ~46% identical at the amino acid sequence level, consistent with sequence variability among the FtxA proteins in *F. alocis* [35]. Taken together, *ftxA* was carried by six of the ten tested strains and is therefore not a universal property of this bacterium. The expression of this gene and its role in *F. alocis* virulence is still not known, yet proteomic characterization of available strains has identified intra-species differences, as well as clustering of FtxA with another six intracellular proteins [36].

## *A. actinomycetemcomitans* and *F. alocis* virulence factors: Contributions to microbial pathogenicity and host immune evasion

### Exotoxins

The different virulence factors expressed by *A. actinomycetemcomitans* are well studied [37] (Supplementary Table S1). Like other gram-negative bacteria, *A. actinomycetemcomitans* releases endotoxins and exotoxins that activate inflammatory response through interaction with the Toll-like receptors 4 (TLR4) [38]. Unique for this bacterium among the inhabitants in the oral microbiota is the expression of two exotoxins. One is LtxA, that is closely associated to disease onset and progression, as shown by a strong correlation to disease onset in carriers of highly leukotoxic variants of *A. actinomycetemcomitans* [24, 25]. LtxA is secreted from the bacterium by a Type I secretion system [39]. This exotoxin induces cell death of human defense cells in an active pro-inflammatory process named pyroptosis [13, 40]. It is well-established that the dysbiotic immune response in pyroptosis is linked to the pathogenesis of periodontitis [41]. These cellular mechanisms also function as a tool for accessing host-derived nutrients for the invading bacteria [42] (Figure 2). The second exotoxin is the cytolethal distending toxin (Cdt), particularly the active unit CdtB, that enters the nucleus of target cells and induces double-strand breaks in chromosomal DNA of proliferating cells [15] (Figure 2). This toxin induces cellular mechanisms involved in the pathogenesis of periodontitis, but its role in disease initiation and progression is unclear [43, 44]. *F. alocis* virulence factors are beginning to be characterized (Figure 1). Most recently, FtxA, has been identified as a putative ~250 kDa exotoxin of *F. alocis* [35], and similar to LtxA, belongs to the large family of RTX proteins, found in both gram-negative and gram-positive bacteria [45]. The activity of FtxA is not yet known; interestingly it appeared to be encoded and expressed only by six of the 10 assessed *F. alocis* strains [35, 36], suggesting potentially different capabilities to modulate host functions by FtxA-expressing strains, as compared to those that do not encode this protein.

### Membrane vesicles

*A. actinomycetemcomitans* outer membrane vesicles (OMVs) can deliver several biologically active virulence factors to host cells, which can modulate the host response (Figure 2). These include Cdt [46], LtxA [47, 48], peptidoglycan-associated lipoprotein (Pal) [49], and the chaperonin GroEL [50]. *A. actinomycetemcomitans* OMVs carry NOD1- and NOD2-active peptidoglycan, which can be internalized into non-phagocytic human cells including gingival fibroblasts [51]. This supports the role of OMVs as triggers of innate immunity. *A. actinomycetemcomitans* OMVs can bind to the classical and mannose-binding lectin (MBL) complement inhibitor, C4-binding protein, by means of the outer membrane protein A1 (OmpA1) [52], which is consistent with an ability of the vesicles to mediate serum protection *in vitro* [53]. The mechanism(s) of how *A. actinomycetemcomitans* OMVs enter and/or delivers cargo into host cells is not entirely clear. The OMVs appear to enter human cells via clathrin-mediated endocytosis [51, 54], but can also fuse with host cell membranes in a cholesterol-dependent fashion [46]. Toxins delivered *via* OMVs can act as adhesins in receptor-mediated endocytosis [55], albeit neither LtxA nor Cdt were required for the OMV uptake by host cells [46, 48]. Hence, despite that LtxA has an apparent surface localization on the *A. actinomycetemcomitans* OMVs, the LtxA receptor LFA-1 is not required for delivering the toxin into the human host cells [39].

Highly purified extracellular vesicles (EVs) released by the *F. alocis* reference strain ATCC 35896 were recently characterized regarding their proteomic content, using in-gel digestion and liquid chromatography-tandem mass spectrometry (LC-MS/MS) [56]. *F. alocis* EVs proteomics revealed 28 proteins, including lipoproteins, autolysins, *F. alocis* complement inhibitor (FACIN), transporter- and metabolism-related proteins, and ribosomal proteins (Figure 1). Interestingly, FtxA, the recently discovered RTX protein family member, [35] according to its GenBank database definition, was identified in the *F. alocis* EVs proteome [56]. Whether FtxA or any of the other EVs proteins might play a role in the observed immunostimulatory effects of the vesicles on human monocytic and oral keratinocyte cell lines [56], and/or in the EV-mediated inhibition of osteogenesis through TLR2 signaling [57] is not presently clear. However, interestingly, as the osteoclastogenic potency of *F. alocis* EVs (Figure 1) was reduced upon treatment with lipoprotein lipase, lipoproteins may contribute to the systemic bone loss via TLR2 [58].

### Complement

The complement cascade is a process known for its antimicrobial role in bacterial opsonization, which targets the clearance and destruction of the organisms by phagocytes and direct cell lysis by forming the C5b-9 membrane attack complex. However, periodontal pathogens developed effective evasion strategies to counteract complement activation. OMVs appear to play a significant role in the ability of *A. actinomycetemcomitans* to evade complement attack. These vesicles serve as a decoy that triggers complement activation through lipopolysaccharide (LPS) and takes in complement components [53]. In turn, LPS of some *A. actinomycetemcomitans* strains (i.e., strain Y4) can bind strongly to C3b, blocking the interaction between complement-derived opsonins with LPS decreasing neutrophils complement-dependent response [59]. Moreover, some of the Omp such as OmpA1 (also known as Omp29 and Omp34), and OmpA2 are important for serum resistance of *A. actinomycetemcomitans* via binding of C4-binding protein, thereby inhibiting the activation of the classical and MBL complement pathways [52]. In response to H_2_O_2_, *A. actinomycetemcomitans* produces Omp100 [60]. Omp100 captures the alternative complement pathway negative regulator, Factor H, and deposits it at the cell surface, modifying and inactivating C3b [61].

The knowledge of *F. alocis'* methods to evade the complement cascade are on the rise. Jusko et al. [62] identified the novel complement inhibitory protein FACIN, which is secreted or expressed on the cell surface and binds to C3, blocking all complement pathways. FACIN has dual importance for *F. alocis* in evading the complement cascade and serving as a cytoplasmic enzyme acetylornithine transaminase involved in arginine catabolism. The authors proposed a mechanism where FACIN binds C3/C3b, yet allows Factor B to bind, then FACIN locks the complex in an inactive state, limiting the C3 convertase as a result (Figure 1).

### Oxidative stress

The dysregulated inflammation and high abundance of hyperactivated neutrophils contribute to the generation of an oxidative-stress enriched environment in the periodontal pocket [34, 63]. Periodontal pathogens develop different survival strategies to detoxify and resist this toxic environment. Depending on the environmental cues, *A. actinomycetemcomitans* activates the oxygen resistance transcription regulator (*oxyR*), which regulates the expression of Omp100 and catalase (KatA) [60] (Figure 2). Catalase aids in the degradation of H_2_O_2_ produced by neutrophils and streptococci [64], protecting *A. actinomycetemcomitans* from oxidative damage. This in turn increases oxygen availability allowing *A. actinomycetemcomitans* to shift from fermentative to respiratory metabolism.

*F. alocis* has been reported to possess virulence factors that contribute to the organism's resistance to oxidative stress. Furthermore, *in vitro*, the growth of *F. alocis* is stimulated under oxidative stress conditions. *F. alocis* reference strain ATCC 35896 encodes an antioxidant enzyme, superoxide reductase FA796 (Figure 1), that reduces superoxide radicals into H_2_O_2_ [65]. *In vitro*, FA796 and the hypothetical protein FA519 are involved in resistance to H_2_O_2_-induced oxidative stress, protection against superoxide radicals, and air exposure, however, the exact mechanisms are unknown. The FA519 protein might confer *F. alocis* the ability to resist both H_2_O_2_ and nitric oxide-induced oxidative stress [66]. Interestingly, the expression of the FA519 genes was significantly enhanced when *F. alocis* was co-cultured with *P. gingivalis*. Gene encoding glutathione peroxidase as well as an alkyl hydroperoxide reductase subunit AhpC are found in the genome of *F. alocis* and may function in clearing H_2_O_2_, however, the partner to the latter mentioned protein (AhpF) is missing in the genome.

## Conclusion and future perspective

The observation that the combined presence of *A. actinomycetemcomitans* and *F. alocis* in the subgingival plaque increases the risk for progression of attachment loss might be explained by the differences in growth requirements and regional nutrient and atmospheric conditions. While *A. actinomycetemcomitans* is facultatively anaerobic and able to colonize the gingiva early in the disease process, *F. alocis* is an obligate anaerobic that will preferentially grow in deeper periodontal pockets. Both species express virulence factors that induce cellular and molecular mechanisms in concordance with the pathogenesis of periodontitis. While the virulence of *A. actinomycetemcomitans* is strongly linked to the expression of its two exotoxins (LtxA and Cdt, Figure 2), we only recently started to unravel the virulence patterns of *F. alocis* (Figure 1). The recent report by Miralda et al. [67] with a detailed characterization of *F. alocis* extending neutrophil lifespan is at odds with the capacity of *A. actinomycetemcomitans* to kill leukocytes. In this study, *F. alocis* reference strain ATCC 35896, which expresses the exotoxin, was responsible for extending neutrophil lifespan. These two contradictory properties may be attributed to different toxins (*i.e*., FtxA and LtxA) of the same toxin superfamily, different expression levels, and differential microbial evasion strategies to overcome neutrophil responses. Here, we reviewed and discussed the virulence factors of *A. actinomycetemcomitans* and *F. alocis* and their pathogenic role in periodontitis (Figure 2). Several open questions arise, like the possible role of FtxA in the pathogenesis of periodontitis, which remains to be evaluated, as well as the possible synergies between FtxA and LtxA. Increased knowledge about the virulence of these two bacteria one by one or together might be of importance for improved risk prediction in the future.

## Author contributions

HO and IS were involved in drafting some sections of the manuscript, designing Supplementary Table S1, Figure 1 (IS), and Figure 2 (HO). GB, JO, AJ, and SMU were involved in the conceptual idea, writing, and critical revision of the manuscript as well as obtaining funding.

## Funding

This work was supported by the NIH-National Institute of Dental and Craniofacial Research (NIDCR) DE024509 and DE014615 (SMU), by Ruth L. Kirschstein National Research Service Award by the NIDCR F31DE027585 (HO), by TUA grants from Region Västerbotten, Sweden [7002667 (AJ) and 7003193 (JO)], and by grants from Insamlingsstiftelsen, Medical Faculty, Umeå University (JO and AJ).

## Conflict of interest

The authors declare that the research was conducted in the absence of any commercial or financial relationships that could be construed as a potential conflict of interest.

## Publisher's note

All claims expressed in this article are solely those of the authors and do not necessarily represent those of their affiliated organizations, or those of the publisher, the editors and the reviewers. Any product that may be evaluated in this article, or claim that may be made by its manufacturer, is not guaranteed or endorsed by the publisher.
