# Supplementary material for: Aggregatibacter actinomycetemcomitans and Filifactor alocis: Two exotoxin-producing oral pathogens
Source: Front Oral Health. 2022 Aug 15;3:981343. doi: 10.3389/froh.2022.981343 (PMC9420871; doi:10.3389/froh.2022.981343)
Supplement: Supplementary file 1 [file Table_1.DOCX]

Supplemental Table 1. Virulence factors of *A. actinomycetemcomitans* and *F. alocis*

| **Virulence Factor** | **Functions and immune evasion** | **Refs** |
| --- | --- | --- |
| **Virulence determinants in *A. actinomycetemcomitans*** | | |
| **Leukotoxin A** | Important for initial binding to host cells. Induces cell death of leukocytes and release of IL-1β and IL-8 by monocytes/macrophages. Induces rapid release of lysosomal enzymes and MMPs from PMNs. | (1-5) |
| **Cytolethal Distending Toxin** | Genotoxin, CdtB (active unit) has DNase and phosphatase activity. Induces cell-cycle arrest and apoptosis in T cells, monocytes, and gingival epithelial cells. Induces the release of IL-1β, IL-6, and IL-8 by PBMCs, monocytes, and macrophages. At high concentrations it ↓IL-12 and IL-10 vs ↑IL-1β and TNFα. Impairs phagocytosis and NO production by macrophages. | (6-10) |
| **Lipopolysaccharide** | Induces bone resorption, tolerance in macrophages, and release of IL-1α, IL-1β, and MMP9. Stimulates the expression of MIP-1α, MIP-1β, MIP-1γ, RANTES, MIP-2, IP-10, and MCP-1 in macrophages. | (11-17) |
| **Outer Membrane Vesicles** | Vehicle to traffic protein factors into host cells, including microRNAs, exRNAs, and virulence factors CdtB, LtxA, and LPS. ExRNAs promote TNFα production in macrophages and can cross BBB. OMVs can internalize into host cells and act as decoys triggering complement through Omps that binding to C4-binding protein or C3b inhibiting the complement pathways. | (9, 18-22) |
| **Virulence determinants in *F. alocis*** | | |
| **RTX toxin-FtxA** | Uncategorized | (23) |
| ***Filifactor alocis* Complement Inhibitor (FACIN)** | Inhibition of complement pathways and opsonization via C3 binding | (24) |
| **FA796 Protein and FA519 Protein (hypothetical)** | Oxidative stress resistance via conversion of superoxide radicals into H_2_0_2_ (FA796) and resistance to H_2_0_2_ (FA796 and FA519) | (25, 26) |
| **Extracellular Vesicles** | Contain 28 proteins (including FACIN, metabolism-related proteins, glycoproteins, and more) that stimulate pro-inflammatory cytokine secretion and bone resorption by stimulating osteoclastogenesis via TLR2 | (27-29) |
| **Lipoteichoic Acid** | Induces the expression of pro-inflammatory cytokines in human gingival fibroblasts. | (30) |

References

1. Venketaraman, V., Lin, A.K., Le, A.*, et al.*, Both leukotoxin and poly-N-acetylglucosamine surface polysaccharide protect Aggregatibacter actinomycetemcomitans cells from macrophage killing. Microb Pathog (2008) 45:173-180. doi: 10.1016/j.micpath.2008.05.007.

2. Kelk, P., Claesson, R., Chen, C.*, et al.*, IL-1beta secretion induced by Aggregatibacter (Actinobacillus) actinomycetemcomitans is mainly caused by the leukotoxin. Int J Med Microbiol (2008) 298:529-541. doi: 10.1016/j.ijmm.2007.06.005.

3. Balashova, N.V., Shah, C., Patel, J.K.*, et al.*, Aggregatibacter actinomycetemcomitans LtxC is required for leukotoxin activity and initial interaction between toxin and host cells. Gene (2009) 443:42-47. doi: 10.1016/j.gene.2009.05.002.

4. Johansson, A., Aggregatibacter actinomycetemcomitans leukotoxin: a powerful tool with capacity to cause imbalance in the host inflammatory response. Toxins (Basel) (2011) 3:242-259. doi: 10.3390/toxins3030242.

5. Balashova, N., Dhingra, A., Boesze-Battaglia, K., and Lally, E.T., Aggregatibacter actinomycetemcomitans leukotoxin induces cytosol acidification in LFA-1 expressing immune cells. Mol Oral Microbiol (2016) 31:106-114. doi: 10.1111/omi.12136.

6. Shenker, B.J., Hoffmaster, R.H., McKay, T.L., and Demuth, D.R., Expression of the cytolethal distending toxin (Cdt) operon in Actinobacillus actinomycetemcomitans: evidence that the CdtB protein is responsible for G2 arrest of the cell cycle in human T cells. Journal of immunology (2000) 165:2612-2618. doi: 10.4049/jimmunol.165.5.2612.

7. Akifusa, S., Poole, S., Lewthwaite, J.*, et al.*, Recombinant Actinobacillus actinomycetemcomitans cytolethal distending toxin proteins are required to interact to inhibit human cell cycle progression and to stimulate human leukocyte cytokine synthesis. Infect Immun (2001) 69:5925-5930. doi: 10.1128/IAI.69.9.5925-5930.2001.

8. Rabin, S.D., Flitton, J.G., and Demuth, D.R., Aggregatibacter actinomycetemcomitans cytolethal distending toxin induces apoptosis in nonproliferating macrophages by a phosphatase-independent mechanism. Infect Immun (2009) 77:3161-3169. doi: 10.1128/IAI.01227-08.

9. Rompikuntal, P.K., Thay, B., Khan, M.K.*, et al.*, Perinuclear localization of internalized outer membrane vesicles carrying active cytolethal distending toxin from Aggregatibacter actinomycetemcomitans. Infect Immun (2012) 80:31-42. doi: 10.1128/IAI.06069-11.

10. Boesze-Battaglia, K., Walker, L.P., Dhingra, A.*, et al.*, Internalization of the Active Subunit of the Aggregatibacter actinomycetemcomitans Cytolethal Distending Toxin Is Dependent upon Cellugyrin (Synaptogyrin 2), a Host Cell Non-Neuronal Paralog of the Synaptic Vesicle Protein, Synaptogyrin 1. Front Cell Infect Microbiol (2017) 7:469. doi: 10.3389/fcimb.2017.00469.

11. Nishida, E., Hara, Y., Kaneko, T.*, et al.*, Bone resorption and local interleukin-1alpha and interleukin-1beta synthesis induced by Actinobacillus actinomycetemcomitans and Porphyromonas gingivalis lipopolysaccharide. J Periodontal Res (2001) 36:1-8. doi: 10.1034/j.1600-0765.2001.00637.x.

12. Sosroseno, W., Barid, I., Herminajeng, E., and Susilowati, H., Nitric oxide production by a murine macrophage cell line (RAW264.7) stimulated with lipopolysaccharide from Actinobacillus actinomycetemcomitans. Oral Microbiol Immunol (2002) 17:72-78. doi: 10.1046/j.0902-0055.2001.00091.x.

13. Tanabe, S.I., and Grenier, D., Macrophage tolerance response to Aggregatibacter actinomycetemcomitans lipopolysaccharide induces differential regulation of tumor necrosis factor-alpha, interleukin-1 beta and matrix metalloproteinase 9 secretion. J Periodontal Res (2008) 43:372-377. doi: 10.1111/j.1600-0765.2007.01049.x.

14. Chung, J., Choi, M.J., Jeong, S.Y.*, et al.*, Chemokines gene expression of RAW 264.7 cells by Actinobacillus actinomycetemcomitans lipopolysaccharide using microarray and RT-PCR analysis. Mol Cells (2009) 27:257-261. doi: 10.1007/s10059-009-0031-1.

15. Huang, M., Li, H.X., Luo, L.*, et al.*, [Different expression of cytokines induced by Actinobacillus actinomycetemcomitans lipopolysaccharide in monocytes/macrophages from different organs of rabbits]. Zhonghua Kou Qiang Yi Xue Za Zhi (2013) 48:155-160. doi:

16. Mizutani, H., Ishihara, Y., Izawa, A.*, et al.*, Lipopolysaccharide of Aggregatibacter actinomycetemcomitans up-regulates inflammatory cytokines, prostaglandin E2 synthesis and osteoclast formation in interleukin-1 receptor antagonist-deficient mice. J Periodontal Res (2013) 48:748-756. doi: 10.1111/jre.12065.

17. Park, O.J., Cho, M.K., Yun, C.H., and Han, S.H., Lipopolysaccharide of Aggregatibacter actinomycetemcomitans induces the expression of chemokines MCP-1, MIP-1alpha, and IP-10 via similar but distinct signaling pathways in murine macrophages. Immunobiology (2015) 220:1067-1074. doi: 10.1016/j.imbio.2015.05.008.

18. Thay, B., Damm, A., Kufer, T.A.*, et al.*, Aggregatibacter actinomycetemcomitans outer membrane vesicles are internalized in human host cells and trigger NOD1- and NOD2-dependent NF-kappaB activation. Infect Immun (2014) 82:4034-4046. doi: 10.1128/IAI.01980-14.

19. Choi, J.W., Kim, S.C., Hong, S.H., and Lee, H.J., Secretable Small RNAs via Outer Membrane Vesicles in Periodontal Pathogens. J Dent Res (2017) 96:458-466. doi: 10.1177/0022034516685071.

20. Nice, J.B., Balashova, N.V., Kachlany, S.C.*, et al.*, Aggregatibacter actinomycetemcomitans Leukotoxin Is Delivered to Host Cells in an LFA-1-Indepdendent Manner When Associated with Outer Membrane Vesicles. Toxins (Basel) (2018) 10: doi: 10.3390/toxins10100414.

21. Han, E.C., Choi, S.Y., Lee, Y.*, et al.*, Extracellular RNAs in periodontopathogenic outer membrane vesicles promote TNF-alpha production in human macrophages and cross the blood-brain barrier in mice. FASEB J (2019) 33:13412-13422. doi: 10.1096/fj.201901575R.

22. Lindholm, M., Metsaniitty, M., Granstrom, E., and Oscarsson, J., Outer membrane vesicle-mediated serum protection in Aggregatibacter actinomycetemcomitans. J Oral Microbiol (2020) 12:1747857. doi: 10.1080/20002297.2020.1747857.

23. Oscarsson, J., Claesson, R., Bao, K.*, et al.*, Phylogenetic Analysis of Filifactor alocis Strains Isolated from Several Oral Infections Identified a Novel RTX Toxin, FtxA. Toxins (Basel) (2020) 12: doi: 10.3390/toxins12110687.

24. Jusko, M., Miedziak, B., Ermert, D.*, et al.*, FACIN, a Double-Edged Sword of the Emerging Periodontal Pathogen Filifactor alocis: A Metabolic Enzyme Moonlighting as a Complement Inhibitor. Journal of immunology (2016) 197:3245-3259. doi: 10.4049/jimmunol.1600739.

25. Mishra, A., Aja, E., and Fletcher, H.M., Role of Superoxide Reductase FA796 in Oxidative Stress Resistance in Filifactor alocis. Sci Rep (2020) 10:9178. doi: 10.1038/s41598-020-65806-3.

26. Aja, E., Mishra, A., Dou, Y., and Fletcher, H.M., Role of the Filifactor alocis Hypothetical Protein FA519 in Oxidative Stress Resistance. Microbiol Spectr (2021) 9:e0121221. doi: 10.1128/Spectrum.01212-21.

27. Kim, H.Y., Lim, Y., An, S.J., and Choi, B.K., Characterization and immunostimulatory activity of extracellular vesicles from Filifactor alocis. Mol Oral Microbiol (2020) 35:1-9. doi: 10.1111/omi.12272.

28. Kim, H.Y., Song, M.K., Gho, Y.S.*, et al.*, Extracellular vesicles derived from the periodontal pathogen Filifactor alocis induce systemic bone loss through Toll-like receptor 2. J Extracell Vesicles (2021) 10:e12157. doi: 10.1002/jev2.12157.

29. Song, M.K., Kim, H.Y., Choi, B.K., and Kim, H.H., Filifactor alocis-derived extracellular vesicles inhibit osteogenesis through TLR2 signaling. Mol Oral Microbiol (2020) 35:202-210. doi: 10.1111/omi.12307.

30. Yoo, H. J., & Lee, S. H. (2022). Virulence of Filifactor alocis lipoteichoic acid on human gingival fibroblast. *Archives*  *of oral biology*, *135*, 105370. https://doi.org/10.1016/j.archoralbio.2022.105370
